# Primary Dysgerminoma of the Uterine Cervix: A Rare Case Report

**DOI:** 10.1155/2024/6465387

**Published:** 2024-05-31

**Authors:** Biruck Gashawbeza, Bethel Dereje, Ferid A. Abubeker

**Affiliations:** Department of Obstetrics and Gynecology Saint Paul's Hospital Millennium Medical College, Addis Ababa, Ethiopia

## Abstract

**Introduction:**

Primary extragonadal germ cell tumors (EGCTs) are a very rare clinical encounter most commonly reported in males. Among females, the placenta, pelvis, uterus, brain, and mediastinum are the most common extragonadal sites and predominantly display nondysgerminoma histology. In this report, we present a case of a primary cervical dysgerminoma in a young female patient. *Case Report*. An 18-year-old nulligravid woman presented with a 12-month history of vaginal bleeding and discharge. Routine blood tests and serum levels of tumor markers were within normal limits. The chest X-ray was normal. A high-resolution pelvic MRI showed a well-defined lobulated cervicovaginal mass measuring 8 × 6 × 5 cm expanding into the vaginal canal with mild homogenous contrast enhancement. An incisional biopsy was performed vaginally under anesthesia, and histologic findings were consistent with dysgerminoma. A repeat follow-up pelvic MRI was done and showed a reduction in the size of the mass by more than 70%. The patient was treated with 4 cycles of bleomycin, etoposide, and cisplatin chemotherapy. Additional external pelvic beam radiation treatment was administered for a partial response. After 3 months of radiotherapy, a contrast abdominopelvic CT scan showed a recurrent cervicovaginal mass with extension to the pelvic sidewalls. The patient was initiated with ifosfamide, paclitaxel, and cisplatin (ITP) as second-line chemotherapy for a recurrent germ cell tumor but later died from hydronephrosis, chronic anemia, and sepsis.

**Conclusion:**

The uterine cervix is a very unusual site for primary dysgerminoma and can have a very aggressive clinical course. A high index of suspicion and an exhaustive workup are necessary to reach a diagnosis, particularly in a young patient presenting with a cervical lesion.

## 1. Introduction

Primary extragonadal germ cell tumors (EGCTs) are a very rare clinical encounter with an estimated incidence of 1.8-3.4 per million [1]. A widely accepted hypothesis suggests that EGCT originates from the abnormal migration of primordial germ cells (PGCs) from the yolk sac to the genital ridge, resulting in misplacement at different sites, usually in the body's midline. Malignant transformation of these ectopic PGCs leads to tumor development in sites where EGCTs are commonly found [2, 3]. EGCTs are more common among males and most frequently occur in the mediastinum, brain, retroperitoneum, and pineal gland. Among females, the placenta, pelvis, uterus, brain, and mediastinum are the most common extragonadal sites and predominantly display nondysgerminoma histology [4]. Herein, we report a primary cervical dysgerminoma in a young female patient.

## 2. Case Report

An 18-year-old nulligravid woman presented with a 12-month history of vaginal bleeding and discharge. She also had easy fatigability and lightheadedness for the past 6 months. Otherwise, she had no history of urinary or bowel complaints. She is not sexually active. Her family history and past medical history were unremarkable. Routine blood tests and serum levels of lactate dehydrogenase (LDH), *α*-fetoprotein (AFP), and *β*-human chorionic gonadotropin (*β*-hCG) were within the normal limits. The chest X-ray was normal.

**Figure 1:**
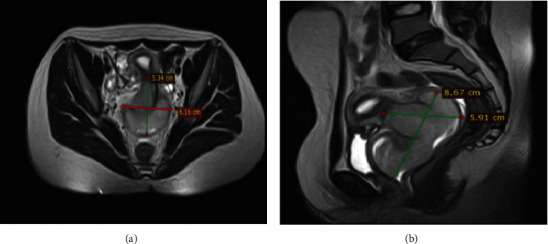
MRI of the primary tumor on (a) axial and (b) sagittal planes showed lobulated 8 *cm* × 6 *cm* × 5 *cm* well-defined homogeneous T2 hyperintense and T1 isointense cervical and vaginal mass expanding in the vaginal canal. Postcontrast imaging also showed a mild homogenous contrast enhancement suggestive of cervicovaginal rhabdomyosarcoma.

A high-resolution pelvic MRI showed a well-defined lobulated cervicovaginal mass measuring 8 × 6 × 5 *cm* expanding into the vaginal canal with mild homogenous contrast enhancement (Figure 1).

**Figure 2:**
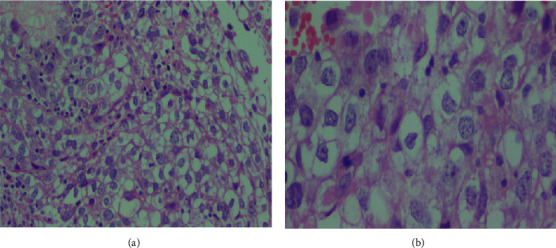
Hematoxylin and eosin (H&E) staining of the histological section at (a) 10x and (b) 15x magnification showed nests of cells with clear cytoplasm and enlarged hyperchromatic nuclei and intervening stroma with lymphocytic infiltrates consistent with cervical dysgerminoma.

With the suspicion of cervicovaginal rhabdomyosarcoma, an incisional biopsy was performed vaginally under anesthesia. Intraoperative findings revealed a well-outlined lower vaginal surface with no clinical evidence of infiltration. However, there was a friable, easily bleeding, gray-to-white mass in the upper 1/4 of the vaginal, and it was difficult to reach the upper margin of the mass. Histologic findings were consistent with dysgerminoma (Figure 2).

**Figure 3:**
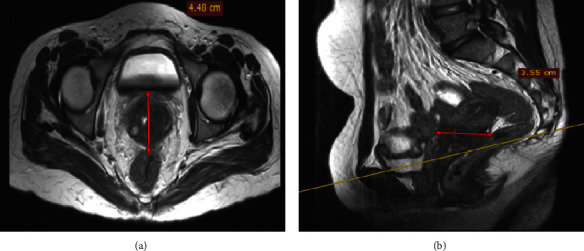
Postchemotherapy MRI on (a) axial and (b) sagittal planes showed an enlarged iliac group of lymph nodes measuring 2 *cm* × 1.7 *cm* and multiple other small lymphadenopathies. The cervix showed a T2 intermediate signal with a 2.3 *cm* × 3.01 *cm* lesion suggestive of a partial radiologic response with RICIST response criteria.

The patient was started on bleomycin, etoposide, and cisplatin (BEP) chemotherapy. Her performance status based on the Eastern Cooperative Oncology Group (ECOG) scoring was three prior to the initiation of chemotherapy. After 4 cycles, she showed clinical improvement with decreased bleeding, weight gain, and overall improved functional status with an ECOG score of one. A repeat follow-up pelvic MRI was done and showed a reduction in the size of the mass by more than 70% (Figure 3). With an impression of partial response, the patient was started on external pelvic beam radiation administered in 23 divided doses after undergoing laparoscopic ovarian transposition.

**Figure 4:**
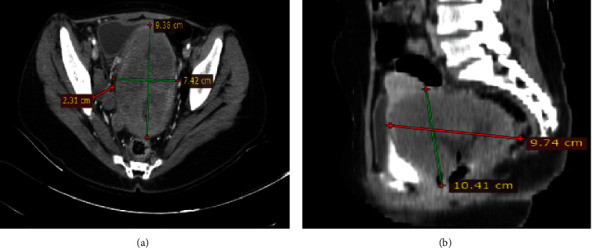
(a) Coronal and (b) sagittal postcontrast CT scans after chemotherapy and pelvic radiation showed a huge abdominopelvic mass extending from the cervicovaginal area and extending to the pelvic side wall, with multiple pelvic lymph nodes, the largest measuring 2 *cm* × 2.5 *cm*, suggesting a recurrent tumor.

After 3 months of radiotherapy, she started to experience back pain radiating to the lower posterior leg and whitish malodorous vaginal discharge. A contrast abdominopelvic CT scan showed a recurrent cervicovaginal mass with extension to the pelvic sidewalls (Figure 4).

A repeat biopsy was taken and showed the same histologic result. The patient was treated with ifosfamide, paclitaxel, and cisplatin (ITP) as second-line chemotherapy for a recurrent germ cell tumor. Though improvement was noted in the first two cycles, the patient subsequently developed worsening of her neuropathic leg pain and abdominal pain. She was started on multivitamins containing vitamins B6 and B12, and treatment with a standing dose of opioid analgesics was continued. Unfortunately, she later succumbed to complications of hydronephrosis, chronic anemia, and sepsis.

## 3. Discussion

Though primary germ cell tumors (GCT) of the ovary account for only 2-5% of ovarian cancer, these neoplasms comprise approximately 20-25% of ovarian malignancy in young women and adolescent girls [5]. Germ cell tumors of the ovary are broadly classified into two large groups: dysgerminoma and nondysgerminoma with significant differences both in natural history and treatment modality. Dysgerminoma is the most common primary malignant ovarian germ cell tumor and generally presents before the age of 40 years, with 75% of cases occurring in the second and third decades of life [6, 7].

In contrast, EGCTs are mostly reported in males and are extremely rare in women [8]. Furthermore, most of the EGCTs among females are of nondysgerminoma histology. National reviews of the cancer registry revealed dysgerminoma accounted for only 11% of EGCTs in the USA and 14% in Germany [4, 9]. Analysis of germ cell tumors in other large population-based cancer registries in Finland and England showed dysgerminoma was observed in 20% and 23%, respectively [10, 11].

Although some studies have identified EGCTs involving the female genital tract and pelvis, involvement of the uterine cervix is extremely rare [7, 9]. Moreover, the cervix is a rare site of metastatic disease from germ cell tumors. Kumar et al. reported a recurrence of ovarian dysgerminoma with cervical metastasis [12]. Otherwise, primary malignant tumors of the uterine cervix are frequently seen in women older than 40 years of age, except for childhood sarcomas arising primarily from the cervix as rhabdoid tumors at an average age of 12.5 years [13, 14]. The uterine and abdominal cavity can also be a primary site for germ cell tumors and dysgerminoma, as described in isolated case reports [15, 16].

Our case was diagnosed with EGCTs of dysgerminoma histology arising from the uterine cervix. Several factors contributed to the diagnostic dilemma in our patient. The very unusual site of the tumor with the indolent course of her symptoms and normal levels of tumor markers made clinical diagnosis difficult. In addition, given the age of the patient, other primary tumors of the cervix were not initially entertained. Further immunohistochemistry analysis would have been helpful for confirmation of the diagnosis and would reliably distinguish between germ cell tumor subtypes [3, 7]. However, immunohistochemistry was not performed due to resource limitations within the healthcare facility as well as due to financial constraints stemming from the patient's inability to afford the test from elsewhere.

One case report [12] described a 24-year-old woman who presented with abdominal pain. Imaging studies revealed a right ovarian tumor measuring 8 × 8 *cm*. In contrast to our case, this patent had an elevated level of LDH at presentation. She underwent surgery and received 4 cycles of BEP chemotherapy. Approximately 2.5 years after surgery, a follow-up speculum examination showed a globular and friable mass on the anterior lip of the cervix. She was diagnosed with dysgerminoma in the cervix but responded well to 3 additional cycles of BEP chemotherapy and showed complete regression of the tumor during follow-up examinations.

The treatment offered to our patient was also considering the responsive nature of dysgerminoma to chemotherapy and radiotherapy. In general, GCTs are known for favorable outcomes [5, 6]. However, the tumor in our patient showed a very aggressive course, with recurrence within three months of radiation requiring treatment with second-line chemotherapy. Some studies also show the prognosis for patients with EGCT is worse than for patients with gonadal GCT [8, 9].

## 4. Conclusion

The uterine cervix is a very unusual site for primary dysgerminoma and can have a very aggressive clinical course. A high index of suspicion and an exhaustive workup are necessary to reach a diagnosis, particularly in a young patient presenting with a cervical lesion.

## Data Availability

The data supporting the conclusions of this report are included within the manuscript.
